# Giant nonlinear self-phase modulation of large-amplitude spin waves in microscopic YIG waveguides

**DOI:** 10.1038/s41598-022-10822-8

**Published:** 2022-05-04

**Authors:** H. Merbouche, B. Divinskiy, K. O. Nikolaev, C. Kaspar, W. H. P. Pernice, D. Gouéré, R. Lebrun, V. Cros, J. Ben Youssef, P. Bortolotti, A. Anane, S. O. Demokritov, V. E. Demidov

**Affiliations:** 1 Institute of Applied Physics, University of Münster, 48149 Münster, Germany; 2 Physics Institute, University of Münster, 48149 Münster, Germany; 3 Center for Soft Nanoscience (SoN), University of Münster, 48149 Münster, Germany; 4 Unité Mixte de Physique, CNRS, Thales, Université Paris-Saclay, 91767 Palaiseau, France; 5 LabSTICC, UMR 6285 CNRS, Université de Bretagne Occidentale, 29238 Brest, France

**Keywords:** Ferromagnetism, Magnetic properties and materials, Spintronics

## Abstract

Nonlinear self-phase modulation is a universal phenomenon responsible, for example, for the formation of propagating dynamic solitons. It has been reported for waves of different physical nature. However its direct experimental observation for spin waves has been challenging. Here we show that exceptionally strong phase modulation can be achieved for spin waves in microscopic waveguides fabricated from nanometer-thick films of magnetic insulator, which support propagation of spin waves with large amplitudes corresponding to angles of magnetization precession exceeding 10°. At these amplitudes, the nonstationary nonlinear dynamic response of the spin system causes an extreme broadening of the spectrum of spin-wave pulses resulting in a strong spatial variation of the spin-wave wavelength and a temporal variation of the spin-wave phase across the pulse. Our findings demonstrate great complexity of nonlinear wave processes in microscopic magnetic structures and importance of their understanding for technical applications of spin waves in integrated devices.

## Introduction

Dynamic magnetic nonlinearities are known as the source of a large variety of fascinating phenomena, which makes magnetic media a unique model system for basic studies in nonlinear physics^1–3^. Among them, magnetic solitons stable excitations formed due to the competition between nonlinearity and dispersion^4,5^ are particularly interesting from the fundamental point of view and are also promising for technical applications^6–11^. The key nonlinear phenomenon responsible for the formation of propagating solitons is the so-called nonlinear self-phase modulation (SPM). It reveals itself in the enrichment of the spectrum of a wave packet propagating in a dispersive nonlinear medium due to the fast variation of the wave intensity at its edges (see, e.g., Ref.^5^). Although SPM is believed to be the main mechanism for the formation of propagating magnetic solitons, its direct experimental observation has been challenging.

The vast majority of experimental studies of nonlinear magnetization dynamics have been performed by using millimeter-scale systems based on relatively thick films of magnetic insulator yttrium iron garnet (YIG) known for its unprecedented small damping. This small damping allows one to reach nonlinear dynamical regimes at moderate excitation powers and maintain the nonlinear propagation of waves of magnetization (spin waves) over large distances. However, small damping is also known to enhance nonlinear decay instabilities^12–20^, which lead to the saturation of the dynamic susceptibility at relatively small amplitudes and do not allow one to drive the spin system into a strongly nonlinear regime.

A significant breakthrough in the field is caused by the recent advent of high-quality YIG films with nanometer thicknesses^21–23^, which enable downscaling of magnetic systems into the sub-micrometer range^24–26^. The strong quantization of the spectrum of magnetic excitations in such systems allows one to reduce the spectral density of dynamic modes and avoid their spectral degeneracy resulting in the suppression of detrimental decay instabilities ^19,27^. As a result, it becomes possible to achieve magnetization oscillations with very large amplitudes leading to strongly nonlinear dynamics^27^, which is difficult to achieve in other nonlinear media.

Here we show that exceptionally strong SPM can be observed for spin-wave pulses in microscopic out-of-plane magnetized YIG waveguides, where one can achieve amplitudes of spin waves corresponding to angles of magnetization precession exceeding 10 degree. Such large amplitudes cause giant SPM leading to a highly efficient broadening of the spectrum of the spin-wave pulse. In particular, in contrast to low-amplitude pulses, where the wavelength of spin waves stays constant across the pulse, large-amplitude pulses exhibit a continuous variation of the wavelength by more than a factor of five. This effect also leads to temporal variation of the spin-wave phase by an amount significantly exceeding 2π over the pulse duration. These phenomena are important not only for understanding the propagation of strongly nonlinear microscopic-scale wave packets and solitons, but are also crucial for the operation of spin-wave devices relying on the phase of spin waves^28–32^ and can be used to extend their functionality.

## Results

**Figure 1:**
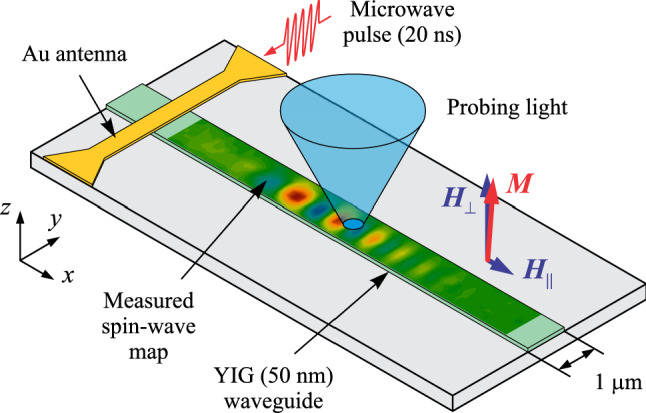
Schematics of the experiment. 20-ns long spin-wave pulses propagate in a 50-nm thick and 1-µm wide YIG waveguide magnetized nearly perpendicularly to its surface. The phase-resolved detection of spin waves is performed by using the micro-focus BLS spectroscopy.

In Fig. 1, we show the schematics of our experiment. We study the propagation of spin-wave pulses in a 1-µm wide waveguide fabricated from a 50 nm thick YIG. The film is characterized by the saturation magnetization µ_0_*M*_S_ = 175 mT and the Gilbert damping parameter α = 1 × 10^–4^. The waveguide is magnetized by the static magnetic field µ_0_*H*_⊥_ = 300 mT applied perpendicular to its surface. Additionally, a small in-plane field µ_0_*H*_||_= 10 mT is applied along the waveguide axis to slightly deflect (deflection angle ≈ 4°) the static magnetization from the normal to the film, which is necessary to enable magneto-optical measurements. The spin-wave pulses are excited by an inductive 400 nm wide Au antenna to which we apply pulses of microwave current with a carrier frequency *f*, a temporal width *w*_T_ = 20 ns, and a rise-/fall-time of about 1.5 ns. The repetition period is chosen to be 500 ns, which guaranties the absence of heating of the sample by intense microwaves with power *P* up to 10 mW.

We analyze the propagation of the excited spin-wave pulses by using time-, space-, and phase-resolved micro-focus Brillouin light scattering (BLS) spectroscopy^33^ (see “Methods” section for details). We focus the probing laser light onto the surface of the waveguide (Fig. 1) and analyze the light scattered from spin waves. The resulting BLS signal is proportional to *A*cos(*φ*), where *A* is the amplitude of the spin wave and *φ* is the phase difference between the microwave excitation signal and the spin wave at the given spatial location. Stroboscopic time-resolved measurements allow us to record two-dimensional phase-resolved spin-wave maps corresponding to the chosen temporal delay with respect to the start of the microwave pulse.

**Figure 2:**
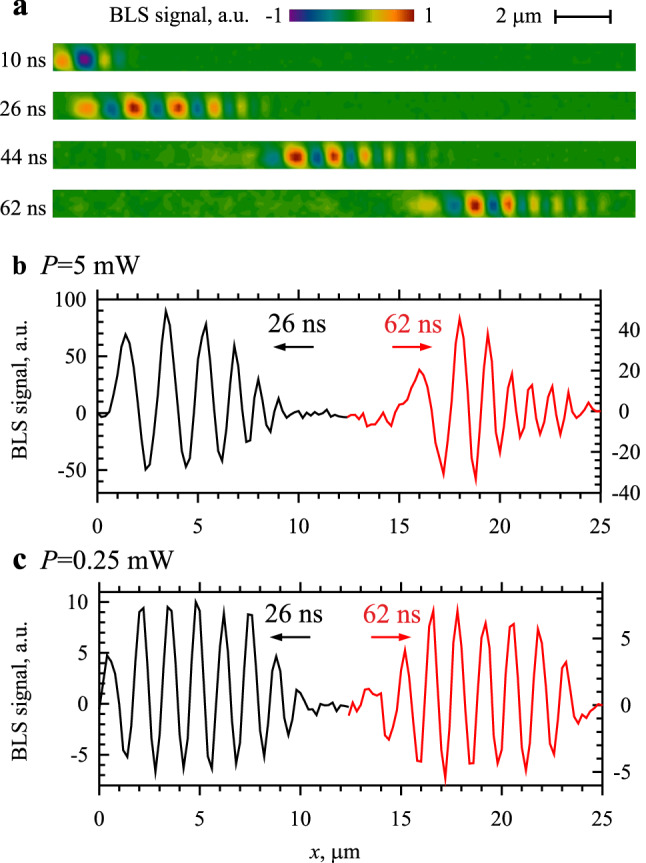
Propagation of nonlinear spin-wave pulses. (**a**) Representative two-dimensional BLS maps visualizing the propagation of the spin-wave pulse. The maps are recorded at *P* = 5 mW and *f* = 4.1 GHz for different temporal delays with respect to the start of the microwave pulse applied to the antenna, as labelled. (**b**) and (**c**) One-dimensional sections of the maps along the waveguide axis for two delays (*t* = 26 and 62 ns) corresponding to the initial and the final stage of the pulse propagation, recorded at *P* = 5 mW (**b**) and 0.25 mW (**c**).

Figure 2a shows representative spin-wave maps recorded at *P* = 5 mW and *f* = 4.1 GHz for different temporal delays. The maps visualize the propagation of the excited spin-wave pulse and allow one to differentiate the characteristic modifications of the pulse profile caused by the joint action of the dispersion and the nonlinearity of the magnetic medium. To facilitate the analysis, we show in Fig. 2b one-dimensional *x*-axis sections of the maps for two delays (*t* = 26 and 62 ns) corresponding to the initial and the final stage of the pulse propagation. Several important features can be seen from these data. First, already at the initial stage, the spin-wave wavelength exhibits a strong variation across the pulse with the short-wavelength components concentrated in its leading part and the long-wavelength components concentrated in the trailing part. Second, during the propagation, the range of involved wavelengths increases: the largest (smallest) period of spatial oscillations at *t* = 62 ns is noticeably larger (smaller) compared to that at *t* = 26 ns. The first observation could be attributed to the effects of group-velocity dispersion (GVD). In the used experimental configuration, the spin waves with smaller wavelengths propagate faster compared to those with larger wavelengths. Since the pulse initially contains different spectral components and small-wavelength components propagate with larger velocities, one can expect the phase modulation seen in Fig. 2b. However, this simple explanation is inconsistent with behaviors observed at smaller microwave powers (see Fig. 2c corresponding to *P* = 0.25 mW). At low power, the spin-wave pulse possesses one dominating wavelength, which remains constant during the propagation. This indicates that GVD alone has a negligible effect on the propagation of the spin-wave pulses. This result can be understood based on the theoretical approach of Ref^34^. Similarly to the approach used in optics^5^, the possible effects of GVD on the propagation of a spin-wave pulse can be characterized using the so-called dispersion time $T_{D} = \frac{{V_{g}^{2} T_{0}^{2} }}{2\pi \left| D \right|}$, where *V*_g_ is the group velocity, *T*_0_ is the pulse width, and $D = \frac{{\partial^{2} \omega}}{{\partial k^{2} }}$ is the dispersion coefficient. This time gives the characteristic propagation time at which dispersion effects become pronounced. From micromagnetic simulations described below, we find *V*_g_ = 0.35 µm/ns, *D* = 1.68 × 10^–2^ µm^2^/ns. Then, for a pulse with a width of 20 ns, the dispersion time is *T*_D_ = 452 ns, which is significantly larger than the time 70–80 ns needed for the pulse to propagate in the studied waveguide. This explains negligible effects of GVD in our experiment. We note, however, that the dispersion effects can become significant for pulses with the width *T*_0_ of several nanoseconds^35^. Overall, the analysis clearly shows that the phase modulation observed in our experiment is not due to GVD, but is rather caused by the nonlinearity of the spin system.

**Figure 3:**
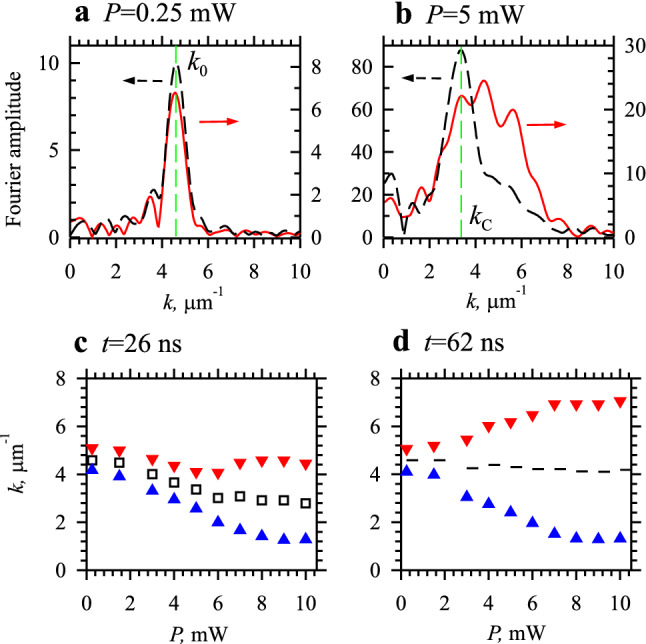
Analysis of the nonlinear phase modulation in the k-space. (**a**) and (**b**) Representative Fourier spectra of the spin-wave pulse obtained at *P* = 0.25 and 5 mW, respectively. Dashed and solid curves show the spectra corresponding to the initial propagation stage (*t* = 26 ns) and the final stage (*t* = 62 ns), respectively. (**c**) and (**d**) Power dependence of the spectral characteristics of the pulse at the initial and the final propagation stage, respectively. Squares−center wavenumber of the spectral peak. Point-up and point-down triangles−the lower and the upper boundaries of the spectrum measured at one half of the maximum amplitude. Dashes−center of mass of the spectrum.

To characterize the observed nonlinear phase modulation quantitatively, we analyze the Fourier spectra of the spin-wave pulses at different propagation stages and at different excitation powers. Figure 3a,b show representative spectra obtained at *P* = 0.25 mW and 5 mW, respectively. The spectra shown by the dashed curves correspond to the initial propagation stage (*t* = 26 ns), while those shown by the solid curves correspond to the final stage (*t* = 62 ns). At low power (Fig. 3a), the spectra are very similar. They exhibit a narrow peak at the wavenumber *k* = *k*_0_ corresponding to the spin-wave wavelength of 1.4 µm (see also Fig. 2c). The amplitude of the peak decreases during the propagation by about 30% due to the damping.

In contrast to the low-power case, at *P* = 5 mW (Fig. 3b), the spectrum of the pulse experiences strong modifications during the propagation. At the initial propagation stage, the spectrum exhibits a pronounced peak at *k* = *k*_C_ < *k*_0_. The peak is noticeably broader compared to the one observed at *P* = 0.25 mW and possesses a tail extending into the region of large wavenumbers. During the propagation, the spectrum broadens further (solid curve in Fig. 3b) exhibiting the dominant growth of the amplitudes of large-*k* (small-wavelengths) spectral components, while the peak amplitude reduces by about 70%.

Qualitatively similar results are obtained at power levels in the range *P* = 0.25–10 mW. At all these powers, the initial-stage spectrum exhibits a pronounced peak, whose center wavenumber *k*_C_ monotonously decreases (squares in Fig. 3c), while its spectral width increases with increasing *P*. After the propagation, the spectrum of the pulse experiences strong further broadening (triangles in Fig. 3d). Note that, at *P* = 10 mW, the spectrum extends over the range *k* = 1.3–7.1 µm^−1^, which corresponds to the variation of the spin-wave wavelength by more than a factor of 5. We emphasize that, such strong effects of SPM have not been observed in other physical systems. For example, in specially designed optical fibers, SPM alone does not allow the generation of optical radiation with a spectrum extending over several octaves in wavelength space (see, e.g., Ref^5^).

## Discussion

**Figure 4:**
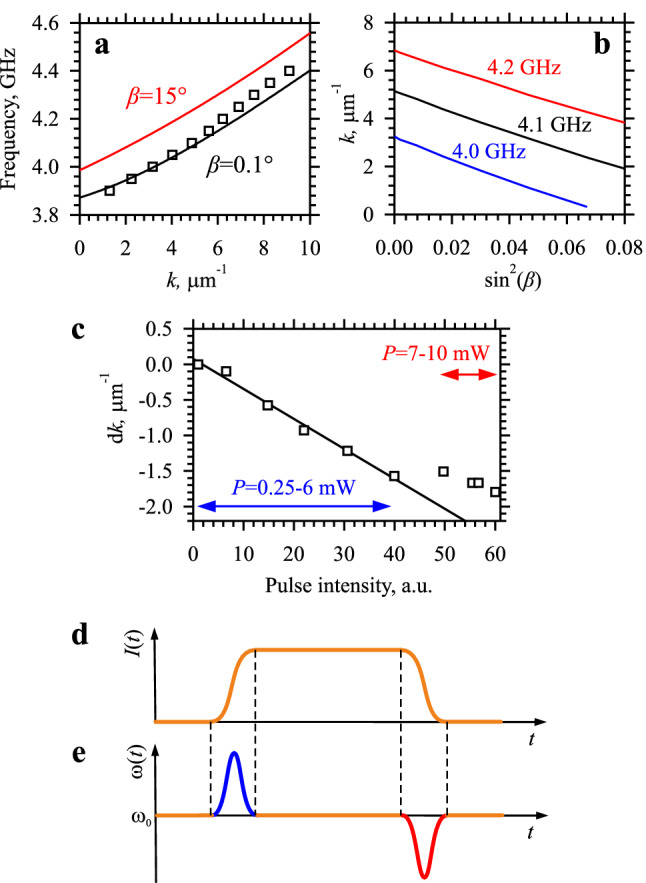
Characterization of the nonlinear spectral shift. (**a**) Curves show the frequency vs wavenumber dependences for spin waves in the studied waveguide obtained from micromagnetic simulations for two different precession angles *β*, as labelled. Squares frequency versus wavenumber dependence measured at *P* = 0.25 mW in the continuous-wave excitation regime. (**b**) Dependences of the wavenumber on $\sin^{2} \left( \beta \right)$ obtained from micromagnetic simulations for different frequencies, as labelled. (**c**) Squares nonlinear reduction of the wavevector d*k* = *k*_C_(*P*)*-k*_0_ corresponding to the initial stage of the pulse propagation as a function of the intensity of the spin-wave pulse detected by BLS. Line linear fit for *P* = 0.25–6 mW. (**d–e**) Schematic illustration of the SPM process. (**d**) Temporal dependence of the wave intensity. (**e**) The resulting modulation of the instantaneous frequency.

In part, the observed behaviors can be associated with the nonlinear shift of the dispersion spectrum of spin waves^3,36,37^. This shift is caused by the reduction of the static component of the magnetization *M*_ST_ with the increase of the amplitude of the magnetization precession: $M_{ST} = \sqrt {\left( {M_{S}^{2} - m^{2} } \right)} \approx M_{S} - \frac{1}{2}M_{S} {\text{sin}}^{2} \left( \beta \right)$, where *M*_S_ is the saturation magnetization, *m* is the amplitude of the dynamic magnetization, and *β* is the precession angle. In the out-of-plane magnetization geometry, the decrease in *M*_ST_ results in an increase of the internal magnetic field *H*_int_ = *H*_⊥_*−M*_ST,_ which leads to a shift of the dispersion spectrum of spin waves towards higher frequencies^36,37^. To quantify the nonlinear shift, we calculate the amplitude-dependent dispersion spectra for spin waves in our waveguide (curves in Fig. 4a) by using the micromagnetic simulation software MuMax3 (Ref.^38^) and the approach developed in Ref.^17^ (see “Methods” section for details). As seen from the data of Fig. 4a, the small-amplitude (*β* = 0.1°) dispersion curve coincides well with that obtained from BLS measurements at low excitation power *P* = 0.25 mW (symbols in Fig. 4a). As expected, the increase of the precession angle results in the shift of the dispersion curve to higher frequencies (see the curve for *β* = 15° in Fig. 4a), while the wavenumber at the given frequency varies nearly linearly with $\sin^{2} \left( \beta \right)$(Fig. 4b).

The results of the simulations allow us to determine the amplitude of the magnetization precession in our experiment. In Fig. 4c, we plot the dependence of the nonlinear reduction of the wavenumber d*k* = *k*_C_(*P*) − *k*_0_ corresponding to the initial stage of the pulse propagation as a function of the intensity of the pulse detected by BLS, which is proportional to $\sin^{2} \left( \beta \right)$. In agreement with the simulations, this dependence is linear in the range *P* = 0.25–6 mW. The deviation from the linear dependence at *P* > 7 mW can be associated with the increasing complexity of the dynamic nonlinear processes, which will be discussed below. By taking the maximum observed d*k*≈1.8 µm^−1^, we derive from the data of Fig. 4b the experimentally achieved precession angle *β* = 12°.

We emphasize that during the propagation, the amplitude of the spin-wave pulse decreases due to damping, which is expected to result in a backward shift of the spin-wave wavenumber toward *k*_0_. This can be responsible for the observed centering of the spectrum at *k*≈*k*_0_ at the final stage of propagation (Fig. 3d). However, the adiabatic variation of the wavenumber during the propagation cannot explain the observed generation of short-wavelength spectral components with *k* > *k*_0_ (see Fig. 3b,d). We associate this generation with the effects of SPM (see, e.g., Ref.^5^), which are illustrated in Fig. 4d,e. When an intense spin-wave pulse propagates in a waveguide, the nonlinear spectral shift discussed above turns out to be nonstationary. Since the intensity of the spin wave $I\sin^{2} \left( \beta \right)$ varies with time in the form of a rectangular pulse (Fig. 4d), the spectral shift remains constant during the pulse plateau and varies rapidly at the leading and trailing edge of the pulse. In this case, one can write for the phase of the spin wave with the carrier frequency *ω*_0_: $\psi\left( t \right) =\omega_{0} t - k\left( {I\left( t \right)} \right)x$, where $k\left( {I\left( t \right)} \right)$ is the time-dependent wavenumber corresponding to the carrier frequency. Then the instantaneous frequency, which is defined as the temporal derivative of the phase, is given by: $\omega\left( t \right) = \frac{d\psi\left( t \right)}{{dt}} =\omega_{0} - \frac{{dk\left( {I\left( t \right)} \right)}}{dt}x$. As follows from this expression and is illustrated in Fig. 4e, the frequency is expected to change at the edges of the pulse due to the rapid variation of $k\left( {I\left( t \right)} \right)$. For example, at the leading edge, the intensity *I*(*t*) increases with time, which leads to a decrease in the wavevenumber *k* (Fig. 4b) and the appearance of a nonzero negative term $\frac{{dk\left( {I\left( t \right)} \right)}}{dt}$. As a result, the instantaneous frequency increases (Fig. 4e). Similarly, the reverse decrease in intensity at the trailing edge results in a decrease of the frequency. Overall, this process can be considered as the generation of new spectral (frequency and wavevector) components at the pulse edges. This is in contrast to stationary nonlinear processes such as four-magnon scattering, which can also be active in the continuous-wave propagation regime.

Assuming a nonlinear shift of the wavenumber of d*k* ≈1 µm^−1^ (Fig. 4c) and a duration of the pulse edge of 1.5 ns (determined by the speed of the microwave modulator), from the above expressions, one can estimate the variation of the instantaneous frequency at the pulse edges of the order of ± 0.1 GHz. Taking into account the slope of the dispersion curve (Fig. 4a), this frequency variation translates into the variation of the wavenumber of about ± 2 µm^−1^, which agrees reasonably well with the width of the *k*-space spectrum in Fig. 3d.

**Figure 5:**
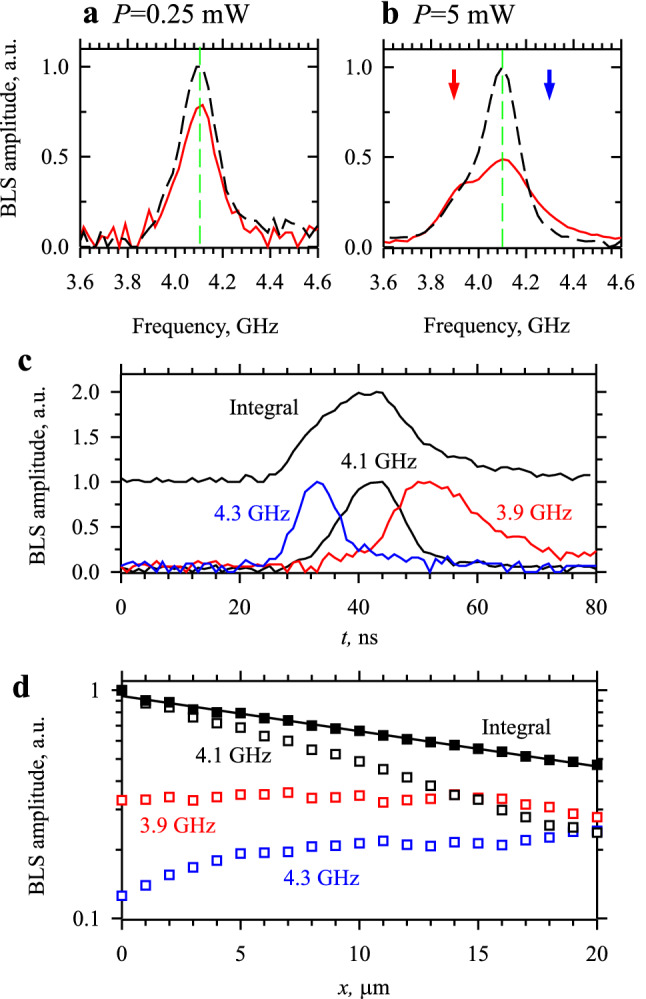
Analysis of SPM in the frequency and spatial domains. (**a**) and (**b**) Frequency spectra of the spin-wave pulses measured at *P* = 0.25 and 5 mW, respectively. Dashed curves show the spectra recorded at *x* = 0, while solid curves show the spectra obtained at *x* = 10 µm. Vertical dashed lines mark the carrier frequency 4.1 GHz. Arrows in (**b**) mark the frequencies of the analyzed side components. (**c**) Normalized temporal profiles of the amplitude envelope *A*(*t*) for the spectral components at the specified frequencies. The profile obtained by integration over the entire frequency spectrum is shown for reference. This profile is shifted by 1 a.u. for clarity. (**d**) Spatial dependences of the amplitude of the main component at 4.1 GHz, side components at 3.9 and 4.3 GHz, as well as the dependence of the total signal integrated over the entire frequency spectrum. Symbols−experimental data. Solid curve−exponential fit.

The analysis of SPM in the *k*-space (Fig. 3) does not allow a direct demonstration of the component generation at the pulse edges. Therefore, to provide an experimental evidence of this phenomenon, we perform a frequency-domain analysis relying on the frequency resolution of the BLS setup. Figure 5a,b show the frequency spectra of the spin-wave pulses measured at *P* = 0.25 and 5 mW, respectively. Dashed curves show the spectra recorded at *x* = 0, while solid curves show the spectra obtained at *x* = 10 µm. As in the case of the *k*-space spectra (Fig. 3a), in the linear propagation regime (Fig. 5a), the frequency spectra are relatively narrow and do not show the generation of new spectral components during propagation. Note here that the width of the spectral peak in Fig. 5a is determined by the limited frequency resolution of the BLS setup and does not reflect the actual width. Despite this limited resolution, the spectra obtained at *P* = 5 mW (Fig. 5b) clearly exhibit an SPM-induced broadening. To understand how the different spectral components are distributed within a pulse, we plot in Fig. 5c the temporal profiles of the amplitude envelope *A*(*t*) for different spectral components. For reference, we also show the profile obtained by integration over the entire frequency spectrum. The data of Fig. 5b clearly show that the component corresponding to the carrier frequency 4.1 GHz dominates in the center of the pulse, while the low-frequency component (3.9 GHz) maximizes at the trailing edge, and the high-frequency component (4.3 GHz) maximizes at the leading edge. This distribution agrees with that expected for the effects of SPM (Fig. 4e).

We emphasize that, in the linear propagation regime (Fig. 5a), the spectrum of the pulse does not contain significant amplitude components at 3.9 and 4.3 GHz. This indicates that their appearance at elevated powers is associated with the nonlinearity of the spin system. Additionally, in the continuous-wave excitation regime, no broadening of the spectrum is observed up to the highest power *P* = 10 mW. This confirms that the efficient nonlinear generation of new spectral components is associated with the nonstationary SPM process.

We now turn to the spatial properties of the nonlinear generation of spectral components. Figure 5d shows the spatial dependences of the amplitude of the main component at 4.1 GHz, side components at 3.9 and 4.3 GHz, as well as the dependence of the total signal obtained by integration over the entire frequency spectrum. The latter dependence exhibits a well-pronounced exponential decay. This indicates that the amplitude-dependent nonlinear damping known for spin waves in in-plane magnetized waveguides^20^ does not play a significant role in the out-of-plane magnetization geometry studied here. This result is not surprising since efficient nonlinear damping requires the existence of spectrally degenerate spin-wave states, which is not the case for out-of-plane magnetized waveguides. Additionally, this geometry lacks the large ellipticity of the magnetization precession required for strong nonlinear damping^17^. The data of Fig. 5d show that instead of the energy loss due to nonlinear damping, there is a continuous SPM-mediated transfer of energy from the initial spectral component to the side components. As a result, the amplitudes of the side components at 3.9 and 4.3 GHz do not decay as the pulse propagates, but even increase noticeably. Note also that these components are already present at the initial stage of pulse propagation (*x* = 0). This indicates that the nonstationary SPM process is active already at the stage of excitation of spin-wave pulses.

**Figure 6:**
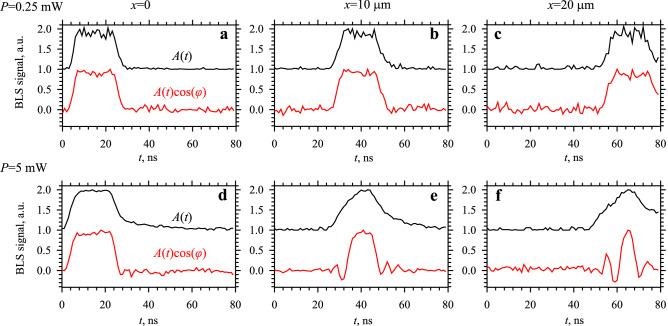
Effects of self-phase modulation in the time domain. Measured temporal profiles of the amplitude envelope of the pulse *A*(*t*) (shifted for clarity) and phase-resolved profiles *A*(*t*)cos(*φ* (*t*)), as labelled. (**a**), (**b**), and (**c**) show the data recorded at *P* = 0.25 mW. (**d**), (**e**), and (**f**) show the data recorded at *P* = 5 mW. (**a**) and (**d**) are recorded at *x* = 0, (**b**) and (**e**) are recorded at *x* = 10 µm, (**c**) and (**f**) are recorded at *x* = 20 µm.

The effects of SPM can be seen particularly clearly in the time domain (Fig. 6). In these measurements, we exploit the ability of the BLS technique to independently measure the amplitude envelope of the pulse *A*(*t*) and the phase-resolved profile *A*(*t*)cos(*φ*(*t*)). Figure 6a–c show these profiles recorded at *P* = 0.25 mW and the propagation distances *x* = 0, 10, and 20 µm. As seen from these data, at low excitation powers, both profiles are very similar. Note that the phase *φ* is the phase difference between the microwave excitation signal and the spin wave at a given spatial location. Therefore, in the absence of phase self-modulation, *φ* depends only on the spatial coordinate and does not depend on time at a given point in space (compare Figs. 6c with 2c). Therefore, the similarity of the *A*(*t*) and *A*(*t*)cos(*φ* (*t*)) profiles is a clear indication that the phase of the carrier spin wave *φ *remains constant across the width of the pulse, as one would expect for a linear weakly dispersive medium. In contrast, the same analysis performed at *P* = 5 mW (Fig. 6d–f) reveals a completely different picture. Close to the antenna (Fig. 6d), the profiles *A*(*t*) and *A*(*t*)cos(*φ* (*t*)) are similar indicating that, initially, the phase is constant across the pulse. However, they start to differ strongly during the pulse propagation. At a distance *x* = 10 µm (Fig. 6e), the profile *A*(*t*)cos(*φ* (*t*)) exhibits oscillations close to the pulse edges indicating that the phase of the spin wave in the edge regions is different from that in the pulse center by at least π/2. This phase modulation becomes even stronger at *x* = 20 µm (Fig. 6f). *A*(*t*)cos(*φ* (*t*)) changes sign several times indicating that the variation of the phase across the pulse width exceeds 2π. On one hand, such phase uncertainty can severely influence the operation of spin-wave devices utilizing the phase of spin waves for information encoding^27–30^. On the other hand, the amplitude-dependent phase modulation can be used for implementation of devices with advanced functionality, e.g., systems for neuromorphic-like computing utilizing nonlinear interference of spin waves^32^.

In conclusion, we have shown that microscopic YIG waveguides support excitation and propagation of intense spin waves characterized by an angle of magnetization precession exceeding 10°. Our results show that, at such large amplitudes, the nonlinearity of the spin system starts to play a decisive role and strongly affects the propagation of spin-wave pulses causing their nonlinear self-phase modulation. This phenomenon reveals itself in the efficient generation of additional spectral components within a broad range of wavelengths and results in the strong variation of the phase across the spin-wave pulse in both space- and time domain. Our results create a base for the understanding of complex nonlinear phenomena in microscopic spin-wave systems and clearly show the importance of these phenomena for practical implementation of integrated spin-wave devices.

## Methods

### Sample fabrication

The 50 nm thick YIG film was grown by liquid phase epitaxy. The 1 µm wide YIG waveguide was defined by e-beam lithography using 300 nm-thick PMMA A4 resist, and Ar ion milling through an Au(10 nm)/Al(60 nm)/Ti(45 nm) hard mask fabricated by lift-off. The sputtered gold layer acted as an oxygen barrier, preserving the YIG stoichiometry from the evaporated Al and Ti layers. After the etching, the remaining Au and Al were removed using selective chemical etching (MF-319 developer for Al and KI/I2 for Au). Au microstrip inductive antenna was defined by e-beam lithography using PMMA A4 resist.

### Micro-focus BLS measurements

All the measurements were performed at room temperature. The probing laser light with a wavelength of 473 nm and a power of 1 mW was focused onto the surface of the waveguide using a high-performance microscope objective lens with magnification of 100 and a numerical aperture of 0.85. The light interacted with the dynamic magnetization in the YIG film resulting in a modulation, whose frequency, intensity, and phase are determined by those of the spin wave at the position of the focal spot. The light carrying the information about the spin wave was collected by the same objective lens, mixed with reference light additionally modulated by the microwave signal used for excitation using an electro-optical modulator, and was finally analyzed with a multi-pass tandem Fabry–Perot interferometer. After processing, the resulting BLS signal is proportional to *A*cos(*φ*), where *A* is the amplitude of the spin wave and *φ* is the phase difference between the microwave excitation signal and the spin wave at the given spatial location. Additionally, we synchronized the detection of the modulated light with the microwave pulses, which enabled stroboscopic time-resolved measurements with a resolution better than 1 ns.

### Micromagnetic simulations

We considered a 1-µm wide and 50-nm thick waveguide with the length *L* = 10 µm discretized into 10 × 10 × 10 nm cells with periodic boundary conditions at the ends. The magnetization dynamics was excited by initially deflecting magnetic moments from their equilibrium orientation in the direction perpendicular to the waveguide axis by an angle, which defined the angle of the excited magnetisation precession* β*. The standard YIG exchange constant of 3.66 pJ/m was used, while the Gilbert damping parameter was set to an artificially small value 10^–12^ to fix the precession amplitude at the chosen level. The initial deflection was spatially periodic with the period *L*/*n* (*n* is an integer number) defining the wavelength of the excited wave. By analysing the free dynamics of magnetization, we determined the frequency corresponding to the given spatial period and obtain the frequency vs wavenumber dependences.

## Data Availability

The datasets generated during and/or analyzed during the current study are available from the corresponding author on reasonable request.
